# Experimental inoculation of CD11c^+^ B1 lymphocytes, CD68^+^ macrophages, or platelet-rich plasma from scrapie-infected sheep into susceptible sheep results in variable infectivity

**DOI:** 10.1099/acmi.0.000155

**Published:** 2020-07-28

**Authors:** Najiba Mammadova, Eric D. Cassmann, S. Jo Moore, Eric M. Nicholson, Justin J. Greenlee

**Affiliations:** ^1^​ Virus and Prion Research Unit, National Animal Disease Center, Agricultural Research Service, United States Department of Agriculture, Ames, IA, USA; ^2^​ Oak Ridge Institute for Science and Education (ORISE) through an interagency agreement between the U.S. Department of Energy (DOE) and the U.S. Department of Agriculture (USDA). ORISE is managed by ORAU under DOE contract number DE-SC0014664

**Keywords:** prion disease, scrapie, B lymphocytes, macrophages, platelets, prions in blood, blood components

## Abstract

Many studies have demonstrated prion infectivity in whole blood and blood components in a variety of transmissible spongiform encephalopathies of livestock and rodents, and variant Creutzfeldt–Jakob disease in humans, as well as an association between pathogenic prion protein (PrP^Sc^) and different immune cells (e.g. follicular dendritic cells, T and B lymphocytes, monocytes and tingible body macrophages). To further investigate the role of various blood components in prion disease transmission, we intracranially inoculated genetically susceptible VRQ/ARQ and ARQ/ARQ sheep with inocula composed of CD11c^+^ B1 lymphocytes, CD68 +macrophages, or platelet-rich plasma derived from clinically ill sheep infected with the US no. 13–7 scrapie agent. At the completion of the study, we found that VRQ/ARQ and ARQ/ARQ sheep inoculated with CD11c^+^ B1 lymphocytes and CD68^+^ macrophages developed scrapie with detectable levels of PrP^Sc^ in the central nervous system and lymphoreticular system, while those inoculated with platelet-rich plasma did not develop disease and did not have detectable PrP^Sc^ by immunohistochemistry or enzyme immunoassay. This study complements and expands on earlier findings that white blood cells harbour prion infectivity, and reports CD11c^+^ B1 lymphocytes and CD68^+^ macrophages as additional targets for possible preclinical detection of prion infection in blood.

## Introduction

Transmissible spongiform encephalopathies (TSEs) or prion diseases are a group of naturally occurring neurodegenerative disorders that include scrapie in sheep and goats, bovine spongiform encephalopathy (BSE), chronic wasting disease (CWD) in cervids and Creutzfeldt–Jakob disease (CJD) in humans. The conformational conversion of the ubiquitously expressed host-encoded cellular prion protein, PrP^C^, to the pathogenic isoform, PrP^Sc^, and its subsequent accumulation throughout the central nervous system (CNS) is widely known as the characteristic molecular event in TSEs. In classical scrapie and other TSEs, there is a long asymptomatic period between infection and the development of neurological clinical signs, during which there is often progressive replication and accumulation of PrP^Sc^ within the lymphoreticular system (e.g. ileal Peyer’s patches, tonsil, spleen and retropharyngeal and mesenteric lymph nodes) [1–5]. During this time, PrP^Sc^ is detectable within lymphoid tissues for months prior to being detectable in the brain, acting as a significant reservoir of prion infectivity to the environment long before the onset of clinical manifestations of the disease [6]. In line with this, recirculating leukocytes within the lymphoreticular system are probable candidates for uptake, sequestration and dissemination of PrP^Sc^ during disease. Indeed, following some of the first evidence of PrP^Sc^ accumulation in lymphoid organs of scrapie-infected animals [1–5, 7–9], a number of studies have demonstrated prion infectivity in whole blood and blood fractions in a variety of TSEs in livestock and rodents, and variant CJD in humans [10–22], as well as an association between PrP^Sc^ and immune cells [e.g. follicular dendritic cells (FDCs), B lymphocytess and tingible body macrophages] [9, 23–26].

To further investigate the role of various blood components in prion disease transmission, we intracranially (IC) inoculated genetically susceptible VRQ/ARQ and ARQ/ARQ sheep with inocula composed of CD11c^+^ B1 lymphocytes, CD68+macrophages, or platelet-rich plasma derived from clinically ill sheep infected with the US no. 13–7 scrapie agent [27]. At the completion of the study, we found that VRQ/ARQ and ARQ/ARQ sheep inoculated with CD11c^+^ B1 lymphocytes and CD68^+^ macrophages developed scrapie with detectable levels of PrP^Sc^ in the CNS and lymphoreticular system (LRS), while those inoculated with platelet-rich plasma did not develop disease. This study confirms earlier findings that white blood cells harbour prion infectivity and provides additional targets (i.e. CD11c^+^ B1 lymphocytes and CD68^+^ macrophages) for possible preclinical detection of prion infection in blood.

## Methods

### Blood donors and recipient sheep

Two different sheep, S300 and B54, were used as blood donors. S300 was an ARQ/ARQ Suffolk sheep that was IC inoculated with brain homogenate prepared from a whole brain derived from a sheep from the second serial passage of the US no. 13–7 scrapie isolate in ARQ/ARQ sheep [28]. B54 was an ARQ/ARK Barbados sheep that was IC inoculated with brain homogenate prepared from a whole brain derived from a sheep from the fifth serial passage of the US no. 13–7 scrapie isolate in ARQ/ARQ sheep [28]. Sheep S300 presented with clinical signs of scrapie 300 days post-inoculation, while donor sheep B54 presented with clinical signs 1099 days post-inoculation.

Recipient sheep of two different genotypes (VRQ/ARQ and ARQ/ARQ) were IC inoculated at 2 months of age with 0.5 ml of inoculum composed of varied blood components (i.e. CD11c^+^ B1 lymphocytes, CD68^+^ macrophages, or platelet-rich plasma) (Table 1) from either donor sheep S300 or B54. The procedure for IC inoculation of lambs has been described previously [29]. All inoculated sheep were housed in biosafety level 2 facilities for 2 weeks following exposure to scrapie. After this period, the sheep were moved to outside pens at the National Animal Disease Center (NADC). The sheep were fed pelleted growth ration and alfalfa hay, and clean water was available *ad libitum*. Non-inoculated control sheep (*n*=5) were kept with the scrapie-free flock at the NADC. Animals were observed daily for the development of clinical signs of neurological disease and were euthanized at the onset of unequivocal clinical signs of disease. Clinical signs of disease included abnormalities in gait and/or stance, and ataxia. Incubation period is reported here as the time from inoculation to the time when unequivocal signs of clinical disease are present. Animals were euthanized at the onset of unequivocal clinical signs of disease, or at the end of the observation period (~5 years).

**Table 1.** Summary of scrapie transmission results in VRQ/ARQ and ARQ/ARQ sheep IC inoculated with varied blood components (i.e. macrophages, B-cells, or platelets) derived from two different donor sheep, B54 and S300. Genotype refers to recipient sheep that were either *PRNP* homozygous (ARQ/ARQ) or heterozygous (VRQ/VRQ) at codon 136. Shading highlights all positive results.

Blood component	Donor	Animal ID	Genotype	Incubation time (days p.i.)	Clinical signs	WB	EIA	IHC CNS	IHC LRS
**CD68^+^** **macrophages**	**B54**	803	VRQ/ARQ	702	POS	POS	POS	POS	POS
		805	VRQ/ARQ	–	NEG	NEG	NEG	NEG	NEG
		963	VRQ/ARQ	–	NEG	NEG	NEG	NEG	NEG
		964	ARQ/ARQ	631	POS	POS	POS	POS	POS
	**S300**	944	VRQ/ARQ	–	NEG	NEG	NEG	NEG	NEG
		960	VRQ/ARQ	652	POS	POS	POS	POS	POS
		967	VRQ/ARQ	–	NEG	NEG	NEG	NEG	NEG
		966	ARQ/ARQ	527	POS	POS	POS	POS	POS
**CD11c^+^ B1 lymphocytes, DCs and DC precursors**	**B54**	941	VRQ/ARQ	–	NEG	NEG	NEG	NEG	NEG
		949	VRQ/ARQ	1303	POS	POS	POS	POS	POS
		961	VRQ/ARQ	616	POS	POS	POS	POS	POS
		938	ARQ/ARQ	–	NEG	NEG	NEG	NEG	NEG
	**S300**	928	VRQ/ARQ	616	POS	POS	POS	POS	POS
		931	VRQ/ARQ	702	POS	POS	POS	POS	POS
		942	VRQ/ARQ	576	POS	POS	POS	POS	POS
		826	ARQ/ARQ	499	POS	POS	POS	POS	POS
**Platelet-rich plasma**	**B54**	812	VRQ/ARQ	–	NEG	NEG	NEG	NEG	NEG
		952	VRQ/ARQ	–	NEG	NEG	NEG	NEG	NEG
		968	VRQ/ARQ	–	NEG	NEG	NEG	NEG	NEG
		*825*	ARQ/ARQ	–	NEG	NEG	NEG	NEG	NEG
	**S300**	932	VRQ/ARQ	–	NEG	NEG	NEG	NEG	NEG
		940	VRQ/ARQ	–	NEG	NEG	NEG	NEG	NEG
		947	VRQ/ARQ	–	NEG	NEG	NEG	NEG	NEG
		*939*	ARQ/ARQ	–	NEG	NEG	NEG	NEG	NEG

EIA, enzyme immunoassay; IHC CNS, immunohistochemistry results in CNS tissues (brain and retina); IHC LRS, immunohistochemistry results in lymphoreticular system [tonsils (palatine and pharyngeal), retropharyngeal and mesenteric lymph node, and spleen]; NEG, PrP^Sc^ was not detected; p.i., post-inoculation; POS, PrP^Sc^ was detected; WB, Western blot.

This study consisted of six groups of four animals (three VRQ/ARQ and one ARQ/ARQ) that were inoculated with a specific blood component (macrophages, B1 lymphocytes, or platelet-rich plasma) derived from donor sheep S300 or B54 (*n*=24 total sheep), as shown in Table 1. At necropsy, duplicate tissue samples were collected and either frozen or stored in 10 % buffered neutral formalin. Specifically, tissues were collected comprising representative sections of the brain, spinal cord, retinas, pituitary, trigeminal ganglia, sciatic nerve, third eyelids, tonsils (palatine and pharyngeal), lymph nodes (retropharyngeal, prescapular and popliteal), spleen, esophagus, forestomaches, intestines, rectal mucosa, thymus, liver, kidney, urinary bladder, pancreas, salivary gland, thyroid gland, adrenal gland, trachea, lung, turbinates, heart, tongue, masseter muscle, diaphragm, triceps brachii, biceps femoris and psoas major.

### Isolation of blood components

Jugular venous blood samples (~200 ml) from donor sheep B54 and S300 were collected either into syringes containing EDTA or into tubes containing acid citrate dextrose as an anticoagulant. Plasma from whole blood in citrate tubes was initially separated by centrifugation (1500 ***g*** for 3 min) and was further centrifuged (5000 ***g*** for 5 min) to prepare platelet-rich plasma (16×10^7^ total cells in 4.8 ml of plasma divided among 9 tubes of 1.6×10^7^ cells from sheep B54, and 15.5×10^7^ total cells in 5.8 ml of plasma divided among 6 tubes of 2.3×10^7^ cells from sheep S300). Blood collected into syringes containing EDTA was divided among four 50 ml centrifuge tubes containing 25 ml of cold 0.075 % phosphate-buffered saline (PBS)/EDTA and centrifuged for 15 min at 3000 r.p.m. at room temperature. Buffy coat cells collected from centrifuged blood samples were then diluted 1 : 1 in PBS/EDTA, layered on 15 ml tubes containing 3 ml of 65 % percoll solution and further centrifuged for 15 min at 3000 r.p.m. without a break. After centrifugation, buffy coat cells were collected from each tube and all tubes from the same animal were combined into one 15 ml tube, resuspended with PBS/EDTA and centrifuged for 7 min at 1700 r.p.m. Buffy coat cells collected from centrifuged blood samples were washed twice with wash buffer [1 % PBS, 0.2 % foetal bovine serum (FBS), 2 mM EDTA] and centrifuged for 7 min at 1700 r.p.m. The cell pellets were then incubated for 10 min on ice with 1 ml of anti-CD8 (6-87-10), 1 ml of anti-CD4 (17D-13) and 1 ml of anti-CD3 (18-106-6). The tube containing the cell pellets and primary antibodies was then filled with wash buffer and centrifuged for 7 min at 1700 r.p.m. In the meantime, a mixture of 20 % goat anti-mouse IgG- or rat anti-mouse IgG1-coupled magnetic beads (Miltenyi Biotec, Bergisch Gladbach, Germany) and 80 % wash buffer was created and added to the tube containing the cell pellets. Additional wash buffer (14 ml) was added to the tube and centrifuged for 7 min at 1700 r.p.m. The cell pellet was then resuspended in ~2 ml of wash buffer and separated using a magnetic cell sorting system (MACS, Miltenyi Biotec). Thereafter, to separate B lymphocytess from macrophages, the negative fraction was further centrifuged and incubated for 10 min on ice with 1 ml of anti-CD68 [1–44]. Goat anti-mouse IgG2b- or goat anti-mouse IgG-coupled magnetic beads and additional wash buffer (14 ml) was again added to the tube and centrifuged for 7 min at 1700 r.p.m. The cell pellet was then resuspended in ~2 ml of wash buffer and separated using a magnetic cell sorting system. The positive fraction consisting of macrophages was collected, counted and separated into six tubes containing PBS to make up 3.16×10^6^ cells 1 ml^−1^ PBS. To separate B1 lymphocytes from B2 lymphocytes, the negative fraction was labelled with anti-CD11c (17-196) and separated using goat anti-mouse IgG- or rat anti-mouse IgG1-coupled magnetic beads and the MACS cell sorting system. The positive fraction consisting of B1 lymphocytes was collected, counted and separated into 10 tubes containing PBS to make up 3.66×10^6^ cells 1 ml^−1^ PBS. All primary antibodies were kindly provided by Dr Alan J. Young.

### Immunohistochemistry

For the detection of PrP^Sc^, slides were stained by an automated immunohistochemistry method using primary antibody F99/F96.7.1 (30) at a concentration of 5 µg ml^−1^, as described previously [31]. Briefly, parafﬁn-embedded sections (4 µm) were rehydrated using xylene, followed by a decreasing ethanol concentration gradient (100, 90–70 %), and a final wash with diH_2_O. Heat-mediated antigen retrieval was performed using citrate buffer (DAKO Target Retrieval Solution, DAKO Corp., Carpinteria, CA, USA) in an autoclave for 30 min. Slides were then stained with an indirect, biotin-free staining system containing an alkaline phosphatase-labelled secondary antibody (*ultra*View Universal Alkaline Phosphatase Red Detection Kit, Ventana Medical Systems, Inc., Tucson, AZ, USA) designed for an automated immunostainer (NexES IHC module, Ventana Medical Systems). Slides were counterstained with Gill’s haematoxylin and bluing agent (Ventana Medical Systems) and then cover slipped. Images were captured using a Nikon DS camera on a Nikon Eclipse 80i microscope. Micrographs were created using a commercial photo-editing system [Adobe Photoshop and Adobe Illustrator (CC); Adobe Systems].

### Enzyme immunoassay (EIA)

A commercially available enzyme immunoassay (HerdChek R, IDEXX Laboratories, Inc., Westbrook, ME, USA) was used to screen for the presence of PrP^Sc^ in brainstem at the level of the obex and the retropharyngeal lymph node (RPLN). Assays were conducted according to the kit instructions, except that the samples were prepared as a 20 % (w/v) tissue homogenate. Cut-oﬀ numbers were determined with a negative control per the kit instructions; values greater than the mean optical density (OD) of negative controls +0.180 were considered to be positive for the purposes of screening samples.

### Western blot analysis

Approximately 0.5 g of brainstem was analysed as described previously, with minor modifications [31]. Samples were homogenized at 4 °C in PBS and digested with proteinase K (PK) for 1 h at 37 °C. PK digestion was stopped using pefabloc (Roche, Indianapolis, IN, USA) to a final concentration of 1 mg ml^−1^. One milligram of homogenate was loaded onto precast sodium dodecyl sulfate (SDS) −12 % polyacrylamide gel electrophoresis (PAGE) gels. SDS-PAGE was performed as described by the manufacturer and the proteins were transferred from the gel to a PVDF membrane with transfer buffer at 35 V for 45 min. The membranes were blocked with 3 % BSA in TBS-T (Tris-buffered saline +0.1 % Tween-20) and either incubated with monoclonal antibody Sha31 for 1 h at room temperature or overnight at 4 °C. A secondary biotinylated sheep anti-mouse secondary antibody (GE Healthcare, Buckinghamshire, UK) at 0.05 µg ml^−1^ and a streptavidin/horseradish peroxidase (HRP) conjugate (GE Healthcare, Buckinghamshire, UK) were used according to the manufacturer’s instructions in conjunction with a chemifluorescent detection system (ECL Plus Detection System, GE Healthcare, Buckinghamshire, UK) and imaged using a multimode scanner (GBOX, Synoptics).

## Results

### Sheep of the VRQ/ARQ and ARQ/ARQ genotypes inoculated with CD11c^+^ B1 lymphocytes and CD68^+^ macrophages developed scrapie

**Fig. 1.**
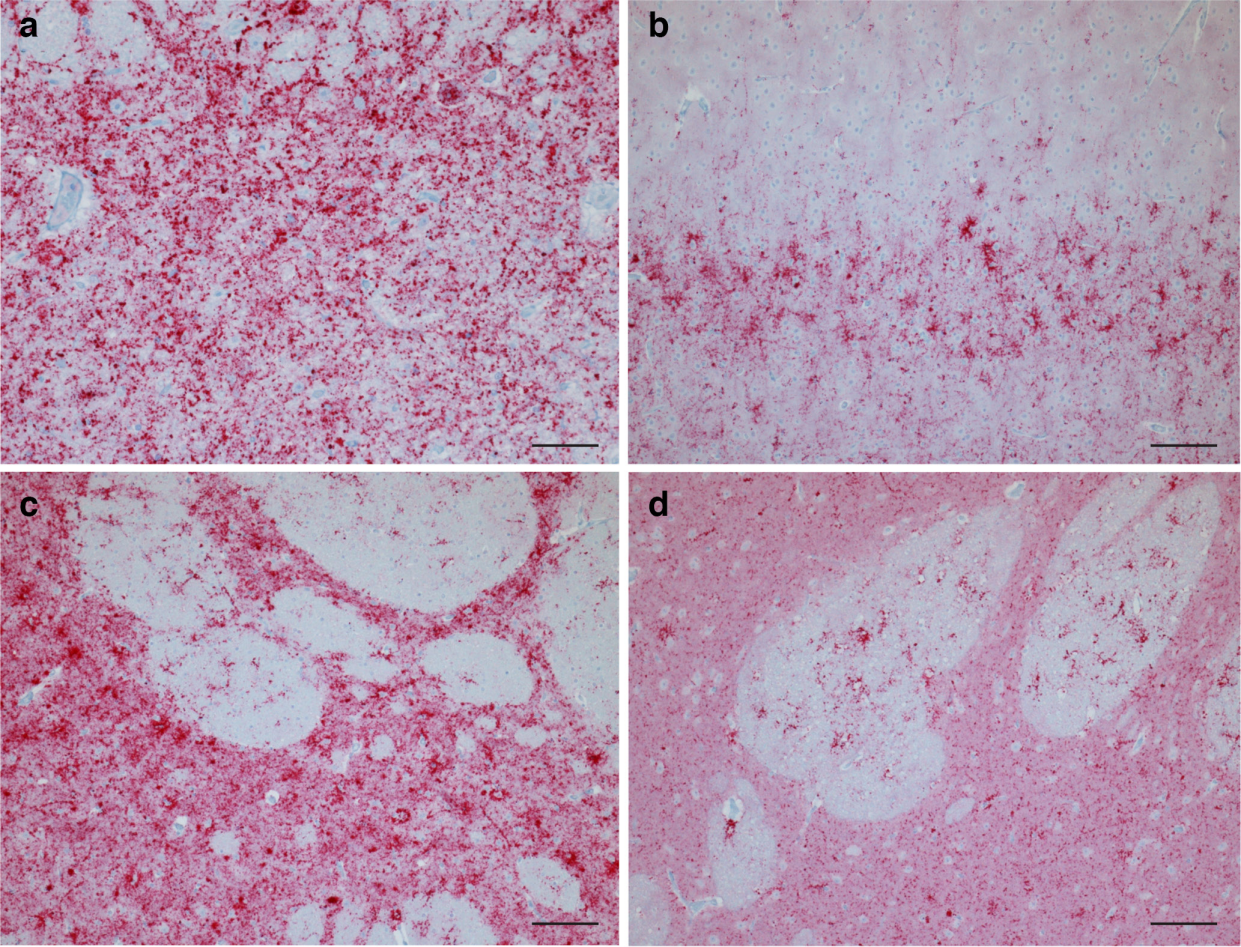
Accumulation of PrP^Sc^ in the CNS. Representative micrographs show PrP^Sc^ immunoreactivity in the brainstem at the level of the obex (a) (scale bar, 50 µm), cerebellum (b) (scale bar, 100 µm), internal capsule of an ARQ/ARQ animal (c) (scale bar, 50 µm), or internal capsule of a VRQ/ARQ animal (d) (scale bar, 100 µm).

**Fig. 2.**
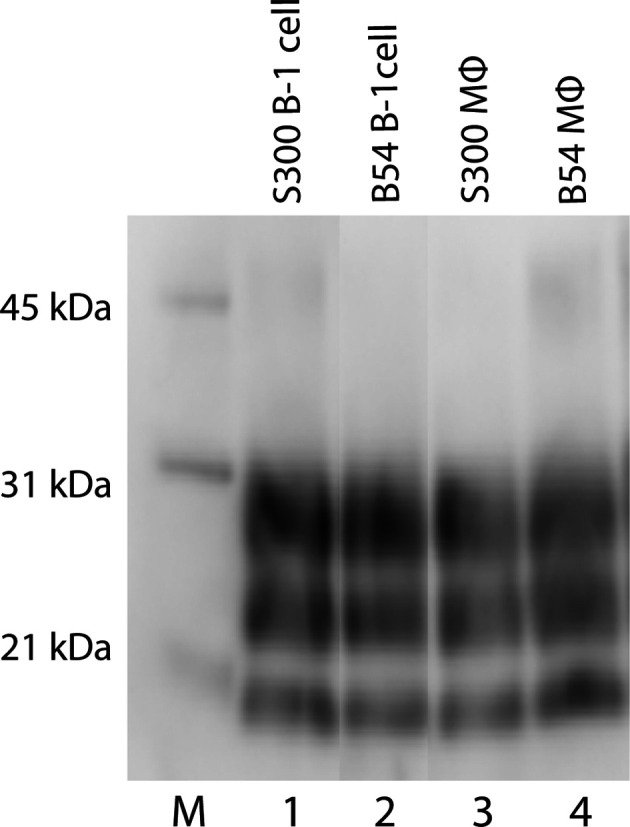
Western blot migration patterns of samples from sheep inoculated with different blood components. Proteinase K digestion of brain homogenates from sheep inoculated with scrapie-infected CD11c^+^ B1 lymphocytes or CD68^+^ macrophages reveals three immunoreactive bands that represent three PrP^Sc^ glycosylation states. Western blot analysis of PrP^Sc^ reveals similar band patterns for scrapie-positive sheep inoculated with CD11c^+^ B1 lymphocytes (lanes 1, 2) and CD68^+^ macrophages (lanes 3, 4) derived from either donor sheep S300 or B54, respectively. To highlight the lanes of interest, blots were cropped from different parts of the same gel. Each lane is representative of all animals in that group. M. molecular weight marker.

To determine whether blood components contained sufficient levels of prion infectivity to cause disease, recipient sheep of two different genotypes (VRQ/ARQ and ARQ/ARQ) were IC inoculated with 0.5 ml of inoculum composed of varied blood components (i.e. CD11c^+^ B1 lymphocytes, CD68^+^ macrophages, or platelet-rich plasma) derived from donor sheep S300 or B54, as shown in Table 1. At the completion of the study, 10 of the 24 animals were determined to be scrapie-positive based on accumulation of pathogenic prion protein (PrP^Sc^) by a combination of immunohistochemistry, Western blot and EIA in CNS and non-CNS tissues [tonsils (palatine and pharyngeal), retropharyngeal and mesenteric lymph node and spleen] (Figs 1 and 2). All 10 animals showed clinical signs of scrapie and had a mean incubation period of 613.4 days post-inoculation, with one outlier (animal 949, which developed clinical signs of scrapie at 1303 days post-inoculation). Of the 10 animals, 4 (2 ARQ/ARQ and 2 VRQ/ARQ) sheep were inoculated with CD68^+^ macrophages and 6 (1 ARQ/ARQ and 5 VRQ/ARQ) sheep were inoculated with CD11c^+^ B1 lymphocytes. None of the animals inoculated with platelet-rich plasma developed disease. There was no noticeable effect of donor genotype on the transmission efficiency of scrapie-infected blood components.

Sheep of the VRQ/ARQ and ARQ/ARQ genotypes inoculated with CD11c^+^ B1 lymphocytes and CD68^+^ macrophages revealed genotype-associated differences in PrP^Sc^ accumulation in the brain.

Immunohistochemistry was used to determine the effect of varied blood components (e.g. macrophages or B1 lymphocytes), donor genotype and/or recipient genotype on PrP^Sc^ deposition in the brain. All scrapie-positive sheep had widespread PrP^Sc^ accumulation throughout the brain. The cell type used in the inoculum (e.g. B1 lymphocytes or macrophages) did not noticeably affect PrP^Sc^ deposition pattern, but different patterns were observed between ARQ/ARQ and VRQ/ARQ sheep, particularly in rostral areas of the brain.

In caudal brain regions, specifically the medulla at the level of the obex, there was marked particulate and intraneuronal immunolabelling and mild to moderate linear and perineuronal immunolabelling (Fig. 1a). In the cerebellum, there was marked particulate immunolabelling in the granular and molecular cell layers, particularly adjacent Purkinje cells, while linear, stellate and intraglial immunolabelling types were commonly observed in the molecular layer (Fig. 1b).

Genotype-associated differences in PrP^Sc^ deposition pattern were most noticeable in rostral brain regions. In particular, there was a difference in the relative amount of immunoreactivity in the grey matter compared to the white matter that was most noticeable in the internal capsule (Fig. 1c, d). In sheep with an ARQ/ARQ genotype, there was more PrP^Sc^ in the grey matter than the white matter (Fig. 1c). In two sheep with the VRQ/ARQ genotype, there was more PrP^Sc^ in the white matter than the grey matter (#803 and #928); however, on average, sheep with the VRQ/ARQ genotype had similar levels of PrP^Sc^ in the white and grey matter at the level of the basal nuclei (Fig. 1d). The pattern of immunoreactivity in the white matter was similar in sheep of both genotypes, with moderate to marked stellate, perivascular, particulate and intraglial immunolabelling types present.

Additionally, the molecular profile of PrP^Sc^ from brainstem homogenates was analysed by Western blot to compare the migration patterns of sheep inoculated with CD11c^+^ B1 lymphocytes versus those inoculated with CD68^+^ macrophages that were positive for the scrapie agent. Western blot analysis revealed a similar banding pattern among sheep inoculated with CD11c^+^ B1 lymphocytes and those inoculated with CD68^+^ macrophages (Fig. 2).

## Discussion

In this study we evaluated whether experimental IC inoculation with B1 lymphocytes, macrophages, or platelet-rich plasma from scrapie-infected sheep into susceptible VRQ/ARQ and ARQ/ARQ sheep would result in infection. Three groups of recipient sheep (six VRQ/ARQ and two ARQ/ARQ animals in each group) were inoculated with 0.5 ml of inoculum composed of 10^6^ macrophages, B1 lymphocytes, or platelets derived from either donor sheep S300 or B54. At the completion of the study, four (two ARQ/ARQ and two VRQ/ARQ) of the eight animals (50%) inoculated with macrophages and six (one ARQ/ARQ and five VRQ/ARQ) of the eight animals (75 %) inoculated with B1 lymphocytes were determined to be scrapie-positive based on accumulation of pathogenic prion protein (PrP^Sc^) by immunohistochemistry, Western blot, and EIA in CNS and lymphoid tissues [tonsils (palatine and pharyngeal), retropharyngeal and mesenteric lymph node and spleen]. PrP^Sc^ was not detected in any of the animals that were inoculated with platelet-rich plasma. This study complements and expands on previously published work that demonstrate scrapie infectivity in whole blood [11, 32] and blood fractions, including peripheral blood mononuclear cells [33, 34], B and T lymphocytess [32, 34] and monocytes [34] (Table 2).

**Table 2.** Summary of scrapie transmission studies of sheep/ovinized mice IV transfused or IC inoculated with different blood components from scrapie-infected sheep. Summary of results from the present study (*). All studies represented in the table used a similar number of cells (~10^6^) in their inocula. Only one study represented in this table utilized a mouse bioassay: ovine VRQ/VRQ PrP transgenic mice (Tg338) were intracerebrally inoculated with classical scrapie isolate PG127 derived from VRQ/VRQ sheep (†).

Blood component	Attack rate	Donor	Recipient	Scrapie strain	Route of inoc.	Reference
PBMCs	4/4	VRQ/VRQ	VRQ/VRQ	natural	IV	[34]
	15/15	VRQ/VRQ	VRQ/VRQ	natural	IV	[33]
CD72^+^ B lymphocytes (pan)	3/3	VRQ/VRQ	VRQ/VRQ	natural	IV	[33]
CD21^+^ B lymphocytes	3/3	VRQ/VRQ	VRQ/VRQ	natural	IV	[33]
CD45^+^ B lymphocytes	4/4	VRQ/VRQ	VRQ/VRQ	PG127	IV	[32]
CD11c B1 lymphocytes DCs, and DC precursors	3/3	ARQ/ARQ	VRQ/ARQ	US 13–7	IC	*
	1/1	ARQ/ARQ	ARQ/ARQ	US 13–7	IC	*
	2/3	ARQ/ARK	VRQ/ARQ	US 13–7	IC	*
	0/1	ARQ/ARK	ARQ/ARQ	US 13–7	IC	*
CD2^+^ (αβ) and γδ T lymphocytes (pan)	1/4	VRQ/VRQ	VRQ/VRQ	natural	IV	[34]
CD4^+^ and CD8^+^ T lymphocytes	2/4	VRQ/VRQ	VRQ/VRQ	PG127	IV	[32]
CD14^+^ monocytes	2/5	VRQ/VRQ	VRQ/VRQ	natural	IV	[34]
	0/4	VRQ/VRQ	VRQ/VRQ	PG127	IV	[32]
CD68^+^ macrophages	1/3	ARQ/ARQ	VRQ/ARQ	US 13–7	IC	*
	1/1	ARQ/ARQ	ARQ/ARQ	US 13–7	IC	*
	1/3	ARQ/ARK	VRQ/ARQ	US 13–7	IC	*
	1/1	ARQ/ARK	ARQ/ARQ	US 13–7	IC	*
Platelet-rich plasma	2/3	ARQ/ARQ	ARQ/ARQ	natural	IV	[33]
Platelet-poor plasma	0/3	ARQ/ARQ	ARQ/ARQ	natural	IV	[33]
Platelets	24/27	VRQ/VRQ†	Tg338†	PG127†	IC	[64]
Platelet-rich plasma	0/3	ARQ/ARQ	VRQ/ARQ	US 13–7	IC	*
	0/1	ARQ/ARQ	ARQ/ARQ	US 13–7	IC	*
	0/3	ARQ/ARK	VRQ/ARQ	US 13–7	IC	*
	0/1	ARQ/ARK	ARQ/ARQ	US 13–7	IC	*

PBMCs, peripheral blood mononuclear cells that include T-lymphocytes, B-lymphocytes, NK cells and monocytes. Genotype, recipient sheep that were either *PRNP* homozygous or heterozygous at codon 136 (ARQ/ARQ, VRQ/VRQ, or VRQ/ARQ respectively) or heterozygous (ARQ/ARK) at codon 171.

### Prion infectivity in CD11c^+^ B1 lymphocytes

Following some of the first evidence of widespread lymphoid accumulation of PrP^Sc^ in scrapie-infected animals [1–5, 7–9], many studies have demonstrated an association between PrP^Sc^ and immune cells [e.g. follicular dendritic cells (FDCs), B lymphocytes and tingible body macrophages] in a variety of TSEs [9, 23–26]. Specifically, B lymphocytes have been associated with the trafficking and dissemination of PrP^Sc^ throughout the lymphoreticular system [25, 35–44]. More recently, Dassanayake *et al*. and Douet *et al*. demonstrated scrapie infectivity following intravenous transfusion of CD72^+^, CD21^+^ and CD45^+^ B lymphocytes prepared from sheep naturally and experimentally infected with classical ovine scrapie [32, 33]. In support, Mathiason *et al*. showed that CD72^+^ B lymphocytes derived from peripheral blood or retropharyngeal lymph nodes can transmit CWD to both native hosts and cervidized mice [26]. The present study expands on these findings in demonstrating that a specific subset of B lymphocytes, CD11c^+^ cells, can transmit scrapie to susceptible VRQ/ARQ and ARQ/ARQ genotype sheep.

Two subpopulations of B lymphocytes have been characterized in several species, including sheep, with apparent distinctions in morphology, function and anatomical distribution [45, 46]: B1 cells that comprise the majority of sheep peripheral blood B-cells [47, 48] and are distinguished by their expression of CD11b and CD11c [47, 48] and B2 cells that are characterized by their expression of CD21 and CD62L [49]. Edwards *et al*. demonstrated a direct association of PrP^Sc^ in blood with the CD11b/CD11c^+^-expressing subset of B lymphocytes [48]. To further support the participation of CD11c^+^-expressing cells in scrapie pathogenesis, a study by Raymond *et al*. revealed that transient *in vivo* depletion of migratory CD11c^+^-expressing cells in Peyer’s patches, mesenteric lymph nodes and the spleens of mice before oral exposure to two scrapie agent strains, ME7 and 139A, blocked the accumulation of PrP^Sc^ in lymphoid tissues and significantly prolonged susceptibility to disease [50]. Functionally, CD11c^+^ B1 lymphocytes are believed to form the first line of defence in an immune response and are a key source of natural IgM antibodies that respond to self- and repetitive antigen in a T-cell-independent manner [45]. While a humoral response is known to be virtually absent in prion infection, many studies have demonstrated that PrP^Sc^ elicits a predominantly IgM response *in vivo* following repeated immunization [51–53]. In line with these data, our study confirms that CD11c^+^ B1 lymphocytes derived from scrapie-infected sheep contain sufficient levels of prion infectivity to cause disease in susceptible VRQ/ARQ and ARQ/ARQ sheep, which has not been previously demonstrated.

The positive sheep in the CD11c+B1 lymphocyte group that received B54-derived inoculum had starkly different incubation periods. The incubation period in sheep #949 was 2.1× longer than that in sheep #961. The discrepancy cannot be accounted for by genotype; both sheep were the VRQ/ARQ genotype. One explanation could be human error during inoculum preparation or inoculation procedures. This is unlikely, given the meticulous procedures regarding the preparation and delivery of inocula. Each tube of CD11c+B1 lymphocytes was treated equally: collection, counting and separation into 10 tubes containing 3.66×10^6^ cells 1 ml^−1^ PBS. The difference between the incubation periods in these animals is less interesting than the long duration of incubation in these sheep. In comparison, sheep with the VRQ/ARQ genotype oronasally inoculated with 0.1 g of 13–7 scrapie have a mean incubation period of 691 days [27]. Sheep with the VRQ/ARQ genotype intracranially inoculated with 0.1 g of 13–7 scrapie have an average incubation period of 428 days (internal unpublished data). The sheep in the present study were also inoculated intracranially, but they have a much longer incubation period. This discrepancy in incubation periods highlights the low levels of PrP^Sc^ associated with CD11c+B1 lymphocytes compared to brain homogenate-derived inocula.

### Prion infectivity in CD68^+^ macrophages

This study reports that experimental IC inoculation of CD68^+^ macrophages from scrapie-infected sheep into susceptible VRQ/ARQ and ARQ/ARQ sheep resulted in transmission to four out of eight animals (50 %). While their specific role in prion disease pathogenesis has not been elucidated, several studies have implicated antigen-presenting cells (i.e. dendritic cells, B-cells and macrophages) as probable candidates for uptake and transport of PrP^Sc^ within the lymphoreticular system [54–60]. In particular, PrP^Sc^ has been demonstrated within the lysosomes of splenic tingible body macrophages, which are predominantly found in germinal centres [9, 61] and in dome macrophages found in Peyer’s patches of scrapie-infected mice and sheep [1, 61, 62]. Maignien *et al*. reported that depletion of macrophages in Peyer’s patches of scrapie- and BSE-infected mice led to an earlier appearance and increased accumulation of PrP^Sc^ in the lymphoreticular system, further suggesting a key role of macrophages in the sequestration and elimination of infectious PrP^Sc^ [63]. However, to the best of our knowledge, the ability of CD68^+^ macrophages to transmit prion disease has not previously been demonstrated.

### Prion infectivity in platelets

Thus far, there is inconsistent evidence associating prion infectivity with platelets. In this report, none of the eight animals inoculated with platelet-rich plasma developed scrapie. The ability of TSE-infected platelets to transmit prion disease has been demonstrated in scrapie-infected ovinized Tg338 mice [64] and sheep [33], BSE-infected sheep [65] and CWD-infected white-tailed deer and cervidized mice [26]. These studies are discrepant with the present work and other observations in hamster scrapie strain 263K [14] and mouse-adapted strains of vCJD or GSS (Fukuoka-1) [36]. Considering that PrP^C^ serves as a template to convert to additional PrP^Sc^, differences in the distribution of PrP^C^ on peripheral blood cells of different species may partly account for the variability in infectivity among rodent species vs bovine, ovine and cervid species [14, 20, 26, 66]. Dassanayake *et al*. demonstrated scrapie infectivity following intravenous transfusion of platelet-rich plasma prepared from sheep naturally infected with classical ovine scrapie [33], which contradicts our results. Additional factors may have contributed to this inconsistency and affected scrapie infectivity across studies, including the scrapie strain of the blood donors; route of inoculation; and/or processing of platelets.

### Genotype-associated differences in PrP^Sc^ accumulation in the brain

Here we report that the cell type used in the inocula (e.g. CD11c^+^ B1 lymphocytes or CD68^+^ macrophages) did not noticeably affect PrP^Sc^ deposition pattern in the brain; however, different patterns were observed between sheep with the ARQ/ARQ and VRQ/ARQ genotypes, predominantly in rostral areas of the brain. Specifically, sheep with the ARQ/ARQ genotype had more PrP^Sc^ in the grey matter than the white matter (most evident at the level of the basal nuclei), while sheep with the VRQ/ARQ genotype had similar levels of PrP^Sc^ in the white and grey matter. The finding of genotype-associated differences in the accumulation of PrP^Sc^ in the grey and white matter of recipient sheep after intracranial inoculation has been observed previously [67]. In order to determine whether the disease phenotype was influenced by inoculum cell type, or host versus recipient genotypes, we constructed immunohistochemical PrP^Sc^ profiles. PrP^Sc^ profiles were generated by scoring the magnitude of distinct morphological immunolabelling types in predefined brain regions, similar to previously described methods [68] (data not shown). Previously published data demonstrated that immunohistochemical PrP^Sc^ profiles can be affected by multiple factors, including the TSE strain, host genotype/breed, incubation times [69–73] and, perhaps, donor genotype. Additionally, significant variation in PrP^Sc^ profiles among animals of the same breed and genotype has also been described [72]. Due to the low sample size in each host/donor genotype group (*n*=1–2 animals that tested positive for the scrapie agent), individual animal variation and/or observer variation, in this particular case, it was unreliable to make comparisons based on PrP^Sc^ profiles.

### Conclusions

In summary, the results reported here confirm that CD11c^+^ B1 lymphocytes and CD68^+^ macrophages isolated from sheep inoculated with the US no. 13–7 scrapie agent contained sufficient levels of prion infectivity to cause disease in VRQ/ARQ and ARQ/ARQ genotype sheep. Inconsistent evidence associating prion infectivity with platelets may be explained by the variability in surface PrP^C^ expression levels of various blood fractions of rodent species vs bovine, ovine and cervid species that could modify a cell subset’s ability to accumulate and/or replicate the prion agent; scrapie strain of the blood donors; route of inoculation; and/or processing of platelets. Whether these blood components are critical to prion pathogenesis has not been elucidated, but this work indicates that prion infectivity in CD11c^+^ B1 lymphocytes and CD68^+^ macrophages was sufficient to cause disease and likely to play a role in haematogenous prion spread. Consistent with this, previous reports have shown that leukoreduction reduced blood-borne prion infectivity [74, 75]. Furthermore, the concentration of prions in whole blood is low [21, 22], and so direct diagnostic tests are unreliable. Enrichment of B1 lymphocytes and macrophages may significantly improve the detection of prion infectivity in blood for enhanced or perhaps early diagnosis. These results provide a starting point for further investigation on the role of CD11c^+^ B1 lymphocytes, CD68^+^ macrophages and platelets in disease progression in sheep and other natural hosts of prion disease.
